# Supplementary material for: Assessing COVID-19 vaccination strategies in varied demographics using an individual-based model
Source: Front Public Health. 2022 Sep 15;10:966756. doi: 10.3389/fpubh.2022.966756 (PMC9521355; doi:10.3389/fpubh.2022.966756)
Supplement: Supplementary file 1 [file Data_Sheet_1.pdf]

Supplementary material

SEIR model, parameters, and their distribution

The SEIR model compartments were implemented with modifications. Our model has the following eight compartments (Figure 1 in the main text):

1. Susceptible - In the beginning, all individuals are susceptible to the disease, except for a chosen number of random individuals that are infected.
2. Latent/Exposed - Susceptible individuals move to the latent compartment after encountering an infectious individual.
3. Infected individuals are either:
    a. Asymptomatic.
    b. Incubating post latent and then Symptomatic.
4. Critical - Some cases are hospitalized and move to a critical stage.
5. Immune - including:
    a. Recovered from a disease.
    b. Vaccinated.
    c. Deceased.

It was assumed that all recovered individuals were fully immune and could not be reinfected. It was also assumed that asymptomatic individuals are half as infectious as the symptomatic individuals and that hospitalized individuals no longer had contacts and could not further infect others. Vaccination was implemented by moving a fraction of individuals directly to the immune compartment. Only individuals with no indication of being sick can be vaccinated. In other words, people in susceptible, latent, incubating post latent, or asymptomatic states can be vaccinated. In our simulation framework, even recovered cases from an asymptomatic infection were not vaccinated, although, in reality, there would probably not be a way to separate them from the susceptibles.

The probability of symptomatic disease given an infection per age group was calculated using a linear interpolation of the estimates by Ma, Qiuyue, et al. (1) to account for a higher resolution of the age groups used in our model. Supplementary Table 1 presents the probability of infected individuals of different age groups to present clinical symptoms.

Age group	0-10	10-20	20-30	30-40	40-50	50-60	60-70	70-80	80+
Probability of symptoms	0.35	0.42	0.49	0.56	0.63	0.7	0.76	0.84	0.87

Supplementary Table 1: Probability of clinical symptoms on infection for age group *i* (y_i).

The delay from disease onset to hospitalization (d_H, in days) was distribured *gamma* (7, 7) (3–5).

Creating a population

Data of two city demographics were incorporated into the model, including age and household size distribution. In particular, the number of individuals in each group was created using a heuristic rejection sampling algorithm based on demographic data from the central bureau of statistics (see “population generation” in the linked code repository).

The generation of households followed town-specific demographic information from the Central Bureau of Statistics. First, lists of the below demographic information were created:

1. Age distribution of the population in the city. The code divides the ages according to the city’s age distribution (https://www.cbs.gov.il/en/subjects/Pages/Population-in-Localities.aspx).
2. The number of houses and the distribution of household sizes (https://www.cbs.gov.il/en/subjects/Pages/Households.aspx).
3. Percentage of households with individuals above the age of 65 (https://www.cbs.gov.il/en/subjects/Pages/Households.aspx).
4. Distribution of the number of children in each household (https://www.cbs.gov.il/en/subjects/Pages/Households.aspx).

Then, households were generated, updating the lists after each household generation (so that the next household was generated based on the distribution of the remaining households).

Calibration of R_e

The model was calibrated to achieve an effective reproduction number R_e at time zero, before the new variant invades, close to 3 (6,7). We used our simulation to find the base infectiousness per contact that corresponds to this value of R_e. We chose a value that allowed for R_e close to 3 in both cities: 0.09 (Supplementary Figure 1). As a sensitivity analysis, we also tested our vaccination strategies with lower base infectiousness of 0.07 and 0.11, corresponding to R_e of about 2.5 and 3.5.

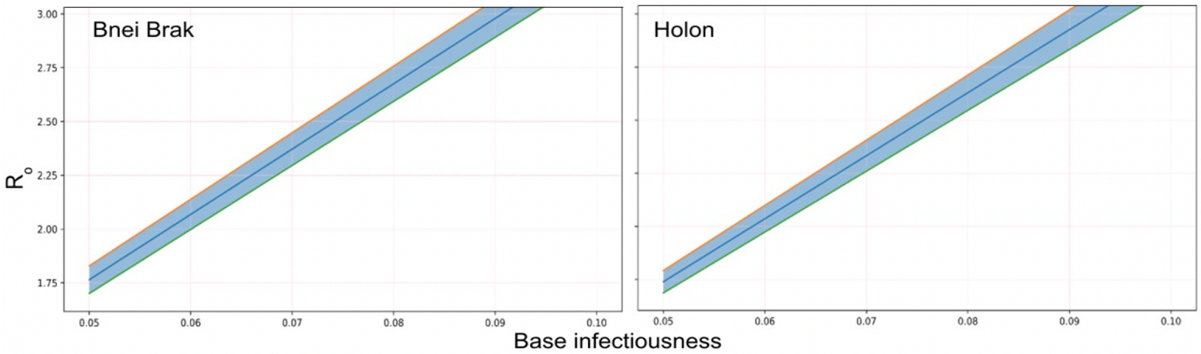

Supplementary Figure 1: R_e as a function of the base infectiousness in the cities of Bnei Brak and Holon. The blue line represents the mean values of the simulation repetitions, and the blue shading represents one standard error from the mean.

Calculation of R_t

The effective reproductive number R_t can be defined as the expected number of new infections caused by one infectious individual at time t. Estimating the effective reproductive number through time involves right censoring. Hence, the instantaneous reproductive number R_t, which is based on past data, is often used in practice to estimate the expected number of new infections at time t, given that all conditions affecting infection remain the same (8). On the other hand, since the instantaneous R_t "looks backward" and uses the information from prior t days for its calculation, it is not meaningful at the beginning of the simulation. Here we use information from the prior 14 days, as only a minor fraction of the infections are still infectious after this period (9). For this reason, we show R_t data only from the 14th day in Figure 2 and Figure S8.

The instantaneous reproductive number is defined as the expected number of secondary infections at time t, divided by the number of infected individuals, each scaled by their relative infectiousness at time t. The relative infectiousness is a function of the generation interval and the time passed since the infection. In our model, we use the *Cori et al.* method to estimate R_t from the simulations (10), as was recommended in *Gostic et al.* (8) and commonly applied in practice (11):

$$R_t = \frac{I_t}{\sum_{s=1}^{t}(I_{t-s} \times w_s)} \qquad (1)$$

Where I_t is the number of infections on day t, and w_s is the relative infectiousness on day t, defined by the generation interval. In other words, this estimator describes the number of new cases on day t, relative to the number (I_{t-s}) individuals who became infected s days before t, weighted by the current infectiousness of individuals that were infected s days ago.

Vaccination strategies

We investigated two additional vaccination strategies to those presented in the main text: *Household* strategy and *All At Once* strategy:

1. *Household* strategy - Aimed to target the households, as this is where individuals spend most of their time. Each day, M households are randomly chosen, and all individuals in the households that belong to the currently prioritized age group are being vaccinated.
2. *All At Once* strategy - Similar to the *Household* strategy, but instead of vaccinating only the current age group, it vaccinates the entire household if at least one household member is in the currently vaccinated age group.

Moreover, we investigated all the strategies without favoring any age group, selecting individuals at random above the minimum age of vaccination.

Supplementary Figures 2 and 3 show the number of infections and hospitalizations using the *Household* and *All At Once* vaccination strategies. Supplementary Figure 4 examines the *General* and *Neighborhood* vaccination strategies, while no age group is prioritized.

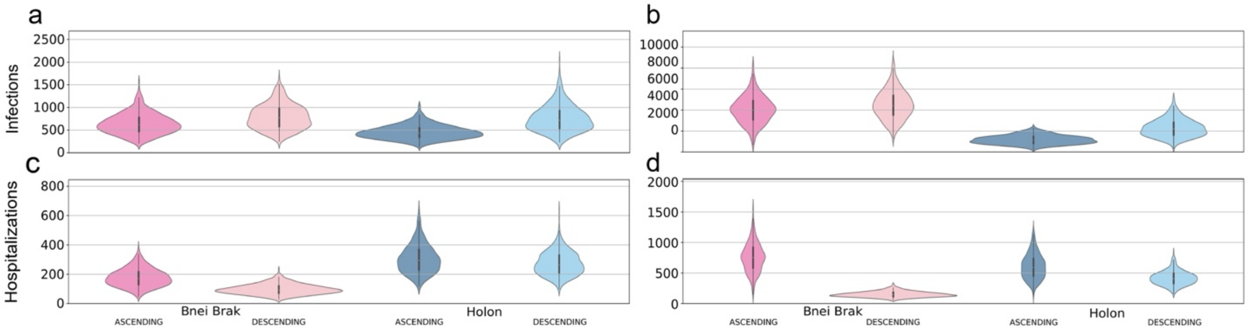

Supplementary Figure 2: Examining the efficiency of the *Household* vaccination strategy, together with the two main NPIs. Each panel shows violin plots of the number of infected (a and b) or hospitalized (c and d) per 100k individuals at the end of 500 simulations. The plots are further stratified by the application of the *Asymptomatic Detection* (a and c) or *Household Isolation* (b and d) interventions.

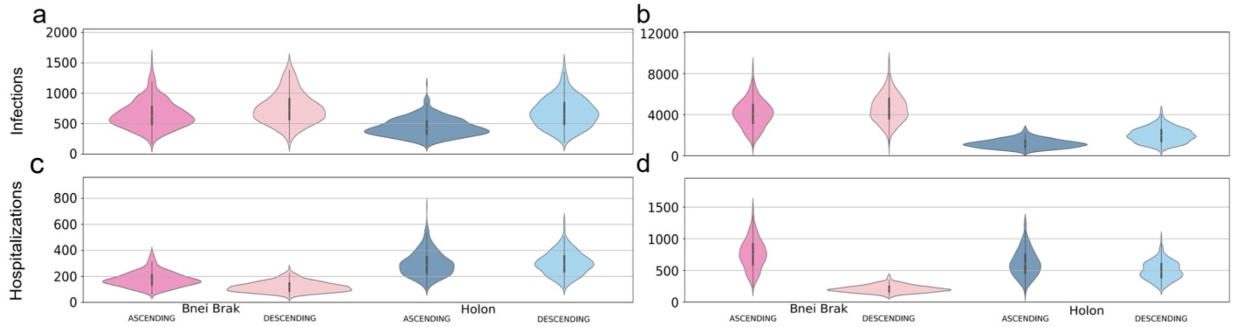

Supplementary Figure 3: Examining the efficiency of the *All At Once* vaccination strategy, together with the two main NPIs. Each panel shows violin plots of the number of infected (a and b) or hospitalized (c and d) per 100k individuals at the end of 500 simulations. The plots are further stratified by the application of the *Asymptomatic Detection* (a and c) or *Household Isolation* (b and d) interventions.

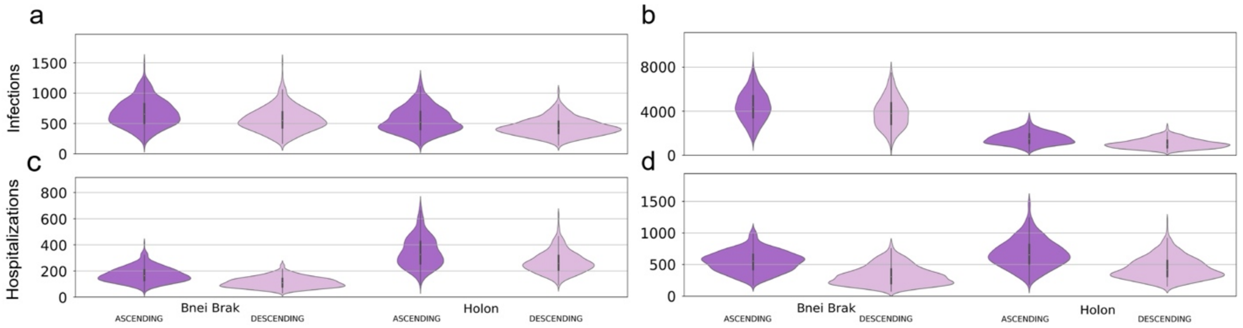

Supplementary Figure 4: Examining the efficiency of the two main NPIs without age prioritization. Each panel shows violin plots of the number of infected (a and b) or hospitalized (c and d) per 100k individuals at the end of 500 simulations. The plots are further stratified by the application of the *Asymptomatic Detection* (a and c) or *Household Isolation* (b and d) interventions.

Sensitivity analyses

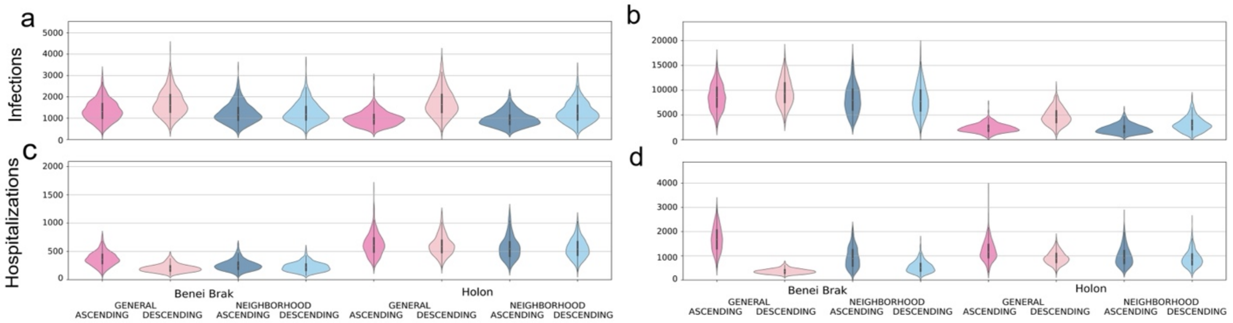

Supplementary Figure 5: Examining the two main NPIs under vaccine efficiency of 80%. Each panel shows violin plots of the number of infected (a and b) or hospitalized (c and d) per 100k individuals at the end of 500 simulations. The plots are further stratified by the application of the *Asymptomatic Detection* (a and c) or *Household Isolation* (b and d) interventions.

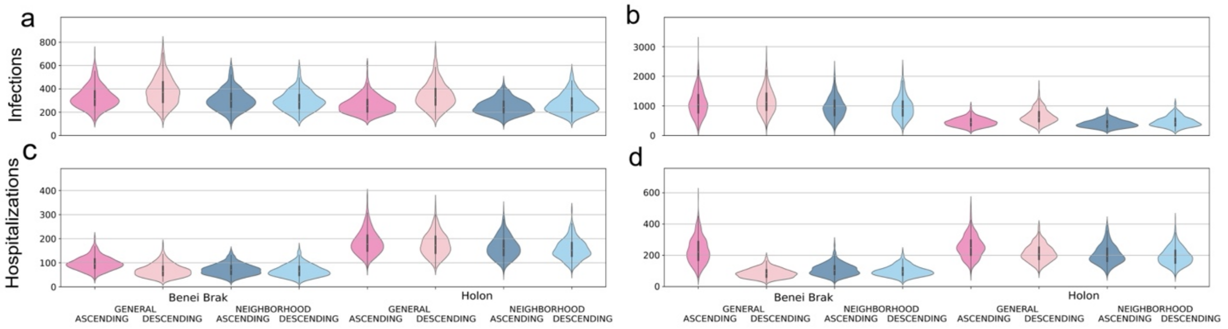

Supplementary Figure 6: Examining the two main NPIs under base infectiousness of 0.07 ($R_e \approx 2.5$). Each panel shows violin plots of the number of infected (a and b) or hospitalized (c and d) per 100k individuals at the end of 500 simulations. The plots are further stratified by the application of the *Asymptomatic Detection* (a and c) or *Household Isolation* (b and d) interventions.

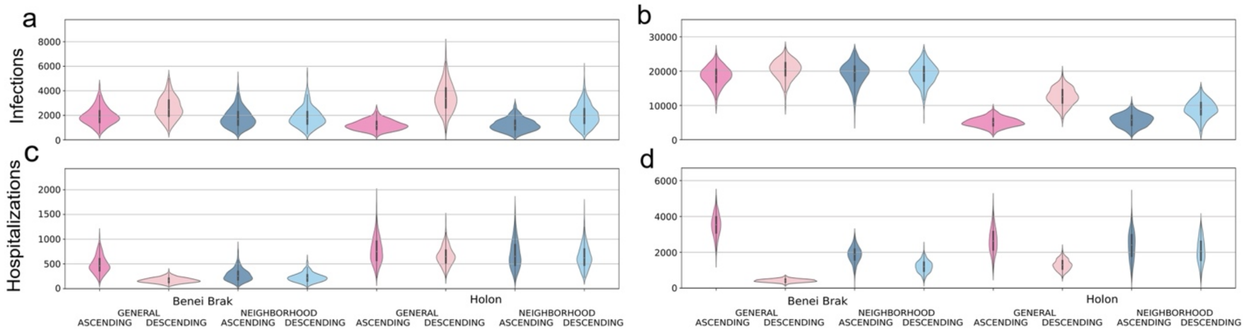

Supplementary Figure 7: Examining the two main NPIs under base infectiousness of 0.11 ($R_e \approx 3.5$). Each panel shows violin plots of the number of infected (a and b) or hospitalized (c and d) per 100k individuals at the end of 500 simulations. The plots are further stratified by the application of the *Asymptomatic Detection* (a and c) or *Household Isolation* (b and d) interventions.

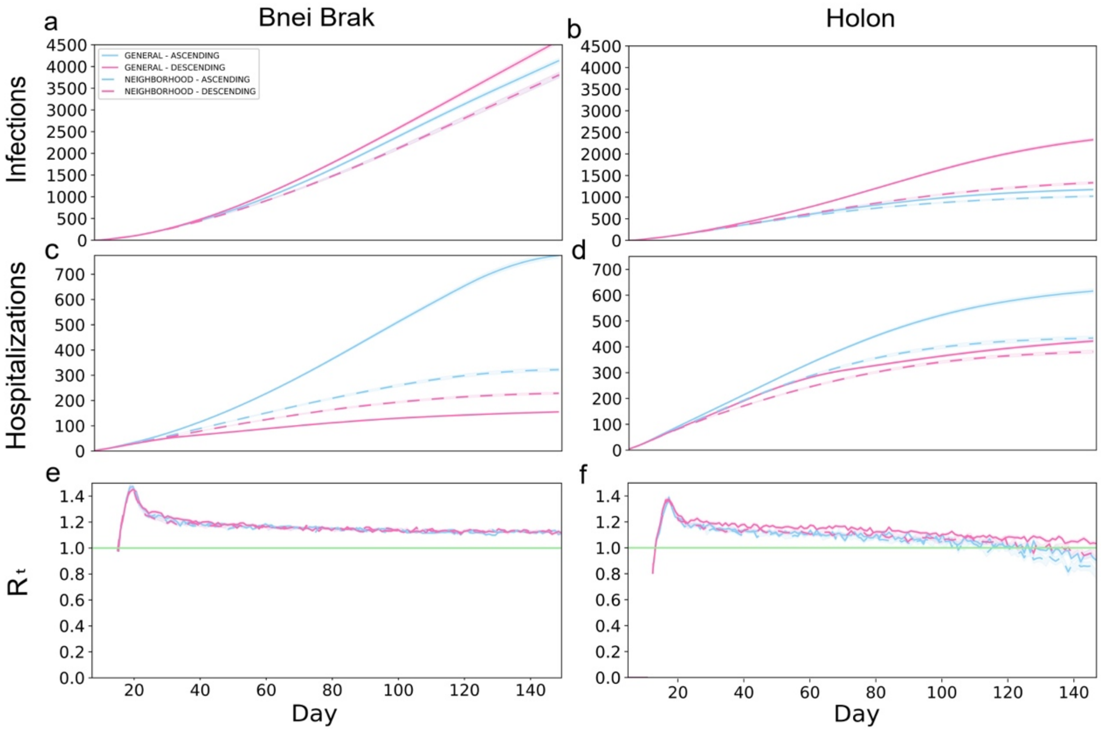

Supplementary Figure 8: Typical outbreak dynamics under different vaccination strategies and using *Household Isolation* intervention. The cumulative number of new infections per 100k (a,b), hospitalizations per 100k (c,d), and R_t (e,f) are shown. The left- and right-hand columns present the results under the demography of Bnei Brak and Holon, respectively. Each panel presents the daily mean of 500 simulations, and the shaded regions around the curves represent the standard error of the mean.

Practical considerations

The complexity of the simulations largely depends on the number of days simulated and the number of infected individuals, as they are tracked over time, their contacts listed, etc. Hence, there is substantial variation in simulation run times. Generally, a single simulation run with characteristics similar to those described in the main text takes a few minutes on a standard personal computer with an Intel® Core™ i7 processor. However, simulation runs can easily be parallelized, as usually many replications of each simulation are needed to account for the stochastic nature of our simulations and to obtain confidence in the calculated statistics.
We ran the simulations on a high-performance cluster containing servers with Intel Xeon Gold 6252 CPUs. The relevant scripts for parallelizing the simulations on such clusters are found with the rest of the code at https://github.com/TAU-COVID19/coderona-virus/tree/vaccination_strategies.

References

1. Ma Q, Liu J, Liu Q, Kang L, Liu R, Jing W, et al. Global Percentage of Asymptomatic SARS-CoV-2 Infections Among the Tested Population and Individuals With Confirmed COVID-19 Diagnosis: A Systematic Review and Meta-analysis. JAMA Netw Open. 2021 Dec 14;4(12):e2137257.
2. Ferguson et al. - 2020 - Report 9 Impact of non-pharmaceutical interventio.pdf.
3. Davies NG, Kucharski AJ, Eggo RM, Gimma A, Edmunds WJ, Jombart T, et al. Effects of non-pharmaceutical interventions on COVID-19 cases, deaths, and demand for hospital services in the UK: a modelling study. Lancet Public Health. 2020 Jul;5(7):e375–85.
4. Linton NM, Kobayashi T, Yang Y, Hayashi K, Akhmetzhanov AR, Jung S mok, et al. Incubation Period and Other Epidemiological Characteristics of 2019 Novel Coronavirus Infections with Right Truncation: A Statistical Analysis of Publicly Available Case Data. J Clin Med. 2020 Feb;9(2):538.
5. Cao B, Wang Y, Wen D, Liu W, Wang J, Fan G, et al. A Trial of Lopinavir–Ritonavir in Adults Hospitalized with Severe Covid-19. N Engl J Med [Internet]. 2020 Mar 18 [cited 2021 May 9]; Available from: https://www.nejm.org/doi/10.1056/NEJMoa2001282
6. D'Arienzo M, Coniglio A. Assessment of the SARS-CoV-2 basic reproduction number, R0, based on the early phase of COVID-19 outbreak in Italy. Biosaf Health. 2020 Jun 1;2(2):57–9.
7. Du Z, Liu C, Wang C, Xu L, Xu M, Wang L, et al. Reproduction Numbers of Severe Acute Respiratory Syndrome Coronavirus 2 (SARS-CoV-2) Variants: A Systematic Review and Meta-analysis. Clin Infect Dis. 2022 Feb 16;ciac137.
8. Gostic KM, McGough L, Baskerville EB, Abbott S, Joshi K, Tedijanto C, et al. Practical considerations for measuring the effective reproductive number, Rt. PLOS Comput Biol. 2020 Dec 10;16(12):e1008409.
9. Singanayagam A, Patel M, Charlett A, Bernal JL, Saliba V, Ellis J, et al. Duration of infectiousness and correlation with RT-PCR cycle threshold values in cases of COVID-19, England, January to May 2020. Eurosurveillance. 2020 Aug 13;25(32):2001483.
10. Cori A, Ferguson NM, Fraser C, Cauchemez S. A New Framework and Software to Estimate Time-Varying Reproduction Numbers During Epidemics. Am J Epidemiol. 2013 Nov 1;178(9):1505–12.

11. Thompson RN, Stockwin JE, van Gaalen RD, Polonsky JA, Kamvar ZN, Demarsh PA, et al. Improved inference of time-varying reproduction numbers during infectious disease outbreaks. Epidemics. 2019 Dec 1;29:100356.
